# Supplementary material for: Structural basis of mitochondrial translation
Source: eLife. 2020 Aug 19;9:e58362. doi: 10.7554/eLife.58362 (PMC7438116; doi:10.7554/eLife.58362)
Supplement: Supplementary file 1. [file elife-58362-supp1.docx]

| Electron Microscope | Titan Krios |
| --- | --- |
| Camera | K2 Summit (counting mode) |
| Voltage | 300 kV |
| Energy filter slit width | 15 eV |
| Nominal Magnification | 130,000 x |
| Calibrated pixel size | 1.05 Å |
| Total exposure | ~30 electrons/Å^2^ |
| Exposure rate | 5.5 electrons/(pixel·s) |
| Number of frames | 20 |
| Defocus range | −0.8 to −2.8 μm |
