# Supplementary material for: Structural basis of mitochondrial translation
Source: eLife. 2020 Aug 19;9:e58362. doi: 10.7554/eLife.58362 (PMC7438116; doi:10.7554/eLife.58362)
Supplement: Supplementary file 2. — The proteins are listed according to the weighted spectra from high to low that was quantified using Scaffold_4.7.5. The pyruvate dehydrogenase complex components and non-mitoribosomal components are shown in italic; LRPPRC and SLIRP are highlighted in yellow. [file elife-58362-supp2.docx]

| Identified Proteins | Accession Number | Molecular Weight |
| --- | --- | --- |
| Leucine-rich PPR motif-containing protein, mitochondrial  OS=Homo sapiens GN=LRPPRC | LPPRC_HUMAN | 158 kDa |
| *Pyruvate dehydrogenase E1 component subunit alpha, somatic form,*  *mitochondrial OS=Homo sapiens GN=PDHA1* | ODPA_HUMAN | 43 kDa |
| 28S ribosomal protein S22, mitochondrial OS=Homo sapiens GN=MRPS22 | G5E9V5_HUMAN | 41 kDa |
| *Dihydrolipoyllysine-residue acetyltransferase component of pyruvate*  *dehydrogenase complex, mitochondrial OS=Homo sapiens GN=DLAT* | ODP2_HUMAN | 69 kDa |
| *Pyruvate dehydrogenase E1 component subunit beta, mitochondrial OS=Homo*  *sapiens GN=PDHB* | ODPB_HUMAN | 39 kDa |
| 39S ribosomal protein L28, mitochondrial OS=Homo sapiens GN=MRPL28 | Q4TT38_HUMAN | 30 kDa |
| 39S ribosomal protein L15, mitochondrial OS=Homo sapiens GN=MRPL15 | RM15_HUMAN | 33 kDa |
| 28S ribosomal protein S9, mitochondrial OS=Homo sapiens GN=MRPS9 | RT09_HUMAN | 46 kDa |
| 28S ribosomal protein S27, mitochondrial OS=Homo sapiens GN=MRPS27 | B4DRT2_HUMAN | 49 kDa |
| *Pentatricopeptide repeat domain-containing protein 3, mitochondrial*  *OS=Homo sapiens GN=PTCD3* | PTCD3_HUMAN | 79 kDa |
| 39S ribosomal protein L37, mitochondrial OS=Homo sapiens GN=MRPL37 | RM37_HUMAN | 48 kDa |
| 28S ribosomal protein S29, mitochondrial OS=Homo sapiens GN=DAP3 | RT29_HUMAN | 46 kDa |
| 28S ribosomal protein S35, mitochondrial OS=Homo sapiens GN=MRPS35 | RT35_HUMAN | 37 kDa |
| 39S ribosomal protein L39, mitochondrial (Fragment) OS=Homo sapiens GN=MRPL39 | C9JG87_HUMAN | 34 kDa |
| 28S ribosomal protein S31, mitochondrial OS=Homo sapiens GN=MRPS31 | RT31_HUMAN | 45 kDa |
| 39S ribosomal protein L1, mitochondrial OS=Homo sapiens GN=MRPL1 | RM01_HUMAN | 37 kDa |
| 39S ribosomal protein L19, mitochondrial OS=Homo sapiens GN=MRPL19 | RM19_HUMAN | 34 kDa |
| 39S ribosomal protein L24, mitochondrial OS=Homo sapiens GN=MRPL24 | RM24_HUMAN | 25 kDa |
| 39S ribosomal protein L44, mitochondrial OS=Homo sapiens GN=MRPL44 | RM44_HUMAN | 38 kDa |
| 39S ribosomal protein L45, mitochondrial OS=Homo sapiens GN=MRPL45 | RM45_HUMAN | 35 kDa |
| 28S ribosomal protein S5, mitochondrial OS=Homo sapiens GN=MRPS5 | RT05_HUMAN | 48 kDa |
| 28S ribosomal protein S7, mitochondrial OS=Homo sapiens GN=MRPS7 | RT07_HUMAN | 28 kDa |
| 39S ribosomal protein L4, mitochondrial OS=Homo sapiens GN=MRPL4 | RM04_HUMAN | 35 kDa |
| 28S ribosomal protein S30, mitochondrial OS=Homo sapiens GN=MRPS30 | RT30_HUMAN | 50 kDa |
| *Pyruvate dehydrogenase protein X component, mitochondrial OS=Homo*  *sapiens GN=PDHX* | ODPX_HUMAN | 54 kDa |
| 39S ribosomal protein L9, mitochondrial OS=Homo sapiens GN=MRPL9 | RM09_HUMAN | 30 kDa |
| 39S ribosomal protein L46, mitochondrial OS=Homo sapiens GN=MRPL46 | RM46_HUMAN | 32 kDa |
| 28S ribosomal protein S2, mitochondrial OS=Homo sapiens GN=MRPS2 | RT02_HUMAN | 33 kDa |
| 28S ribosomal protein S34, mitochondrial OS=Homo sapiens GN=MRPS34 | C9JJ19_HUMAN | 26 kDa |
| 39S ribosomal protein L13, mitochondrial OS=Homo sapiens GN=MRPL13 | RM13_HUMAN | 21 kDa |
| 39S ribosomal protein L21, mitochondrial OS=Homo sapiens GN=MRPL21 | RM21_HUMAN | 23 kDa |
| 39S ribosomal protein L50, mitochondrial OS=Homo sapiens GN=MRPL50 | RM50_HUMAN | 18 kDa |
| 28S ribosomal protein S23, mitochondrial OS=Homo sapiens GN=MRPS23 | RT23_HUMAN | 22 kDa |
| 39S ribosomal protein L16, mitochondrial OS=Homo sapiens GN=MRPL16 | RM16_HUMAN | 28 kDa |
| 28S ribosomal protein S18b, mitochondrial OS=Homo sapiens GN=MRPS18B | RT18B_HUMAN | 29 kDa |
| 39S ribosomal protein L11, mitochondrial OS=Macaca mulatta GN=MRPL11 | F7DTM2_MACMU | 21 kDa |
| 39S ribosomal protein L40, mitochondrial OS=Homo sapiens GN=MRPL40 | F8WBK5_HUMAN | 20 kDa |
| Peptidyl-tRNA hydrolase ICT1, mitochondrial OS=Homo sapiens GN=ICT1 | ICT1_HUMAN | 24 kDa |
| 39S ribosomal protein L38, mitochondrial OS=Homo sapiens GN=MRPL38 | RM38_HUMAN | 45 kDa |
| 28S ribosomal protein S10, mitochondrial OS=Homo sapiens GN=MRPS10 | RT10_HUMAN | 23 kDa |
| 28S ribosomal protein S25, mitochondrial OS=Homo sapiens GN=MRPS25 | RT25_HUMAN | 20 kDa |
| 28S ribosomal protein S26, mitochondrial OS=Homo sapiens GN=MRPS26 | RT26_HUMAN | 24 kDa |
| 39S ribosomal protein L22, mitochondrial OS=Homo sapiens GN=MRPL22 | E7ESL0_HUMAN | 24 kDa |
| Mitochondrial ribosomal protein L43 OS=Pan troglodytes GN=MRPL43 | H2RB51_PANTR | 18 kDa |
| 28S ribosomal protein S11, mitochondrial OS=Homo sapiens GN=MRPS11 | RT11_HUMAN | 21 kDa |
| SRA stem-loop-interacting RNA-binding protein, mitochondrial OS=Homo  sapiens GN=SLIRP | SLIRP_HUMAN | 12 kDa |
| *[Pyruvate dehydrogenase [lipoamide]] kinase isozyme 3, mitochondrial*  *OS=Homo sapiens GN=PDK3* | PDK3_HUMAN | 47 kDa |
| 39S ribosomal protein L17, mitochondrial OS=Homo sapiens GN=MRPL17 | RM17_HUMAN | 20 kDa |
| Mitochondrial ribosomal protein S33 OS=Homo sapiens GN=MRPS33 | A4D1T3_HUMAN | 13 kDa |
| 39S ribosomal protein L3, mitochondrial OS=Homo sapiens GN=MRPL3 | RM03_HUMAN | 39 kDa |
| 39S ribosomal protein L14, mitochondrial OS=Homo sapiens GN=MRPL14 | RM14_HUMAN | 16 kDa |
| Mitochondrial ribosomal protein S28 variant (Fragment) OS=Homo sapiens | Q53G62_HUMAN | 21 kDa |
| 39S ribosomal protein L41, mitochondrial OS=Homo sapiens GN=MRPL41 | RM41_HUMAN | 15 kDa |
| Mitochondrial ribosomal protein L10, isoform CRA_d OS=Homo sapiens GN=MRPL10 | B4DEH0_HUMAN | 25 kDa |
| 39S ribosomal protein L37, mitochondrial OS=Homo sapiens GN=MRPL2 | C9IY40_HUMAN | 24 kDa |
| 28S ribosomal protein S15, mitochondrial OS=Homo sapiens GN=MRPS15 | RT15_HUMAN | 30 kDa |
| 39S ribosomal protein L49, mitochondrial OS=Homo sapiens GN=MRPL49 | RM49_HUMAN | 19 kDa |
| 28S ribosomal protein S6, mitochondrial OS=Homo sapiens GN=MRPS6 | RT06_HUMAN | 14 kDa |
| 39S ribosomal protein L47, mitochondrial OS=Homo sapiens GN=MRPL47 | RM47_HUMAN | 29 kDa |
| 39S ribosomal protein L52, mitochondrial OS=Homo sapiens GN=MRPL52 | RM52_HUMAN | 14 kDa |
| 28S ribosomal protein S14, mitochondrial OS=Homo sapiens GN=MRPS14 | RT14_HUMAN | 15 kDa |
| 39S ribosomal protein L20, mitochondrial OS=Macaca mulatta GN=MRPL20 | F7EZM8_MACMU | 17 kDa |
| 28S ribosomal protein S21, mitochondrial OS=Homo sapiens GN=MRPS21 | RT21_HUMAN | 11 kDa |
| *Mitochondrial transcription factor A OS=Homo sapiens GN=TFAM* | *E5KSU5_HUMAN* | *29 kDa* |
| 39S ribosomal protein L23, mitochondrial OS=Homo sapiens GN=MRPL23 | RM23_HUMAN | 18 kDa |
| 28S ribosomal protein S16, mitochondrial OS=Homo sapiens GN=MRPS16 | A6ND22_HUMAN | 11 kDa |
| 39S ribosomal protein L51, mitochondrial OS=Homo sapiens GN=MRPL51 | RM51_HUMAN | 15 kDa |
| 60S ribosomal protein L18 OS=Homo sapiens GN=RPL18 | RL18_HUMAN | 22 kDa |
| 39S ribosomal protein L54, mitochondrial OS=Homo sapiens GN=MRPL54 | RM54_HUMAN | 16 kDa |
| 39S ribosomal protein L41, mitochondrial OS=Homo sapiens GN=MRPL27 | D6RAN8_HUMAN | 10 kDa |
| 39S ribosomal protein L48, mitochondrial OS=Homo sapiens GN=MRPL48 | B4DN34_HUMAN | 22 kDa |
| 39S ribosomal protein L53, mitochondrial OS=Homo sapiens GN=MRPL53 | RM53_HUMAN | 12 kDa |
| 60S ribosomal protein L7 OS=Homo sapiens GN=RPL7 | RL7_HUMAN | 29 kDa |
| Mitochondrial assembly of ribosomal large subunit protein 1 OS=Homo sapiens GN=MALSU1 | MASU1_HUMAN | 26 kDa |
| 28S ribosomal protein S12, mitochondrial OS=Homo sapiens GN=MRPS12 | RT12_HUMAN | 15 kDa |
| 28S ribosomal protein S18c, mitochondrial OS=Homo sapiens GN=MRPS18C | D6RCM2_HUMAN | 13 kDa |
| 28S ribosomal protein S17, mitochondrial OS=Homo sapiens GN=MRPS17 | RT17_HUMAN | 15 kDa |
