# Supplementary material for: Structural basis of mitochondrial translation
Source: eLife. 2020 Aug 19;9:e58362. doi: 10.7554/eLife.58362 (PMC7438116; doi:10.7554/eLife.58362)
Supplement: Supplementary file 3. — * FSC corrected for the effect of the mask according to 0.143-cutoff criterion ** FSC (masked) according to 0.5-cutoff criterion [file elife-58362-supp3.docx]

| **atomic model** | **APE** | **AP** | **P** | **PE** | **E** | **A/P, P/E** | **P/E** | **RRF** |
| --- | --- | --- | --- | --- | --- | --- | --- | --- |
| **map resolution (Å)*** | **4.0** | **4.0** | **4.5** | **3.7** | **3.5** | **5.0** | **3.8** | **4.0** |
| **CC-volume (map/model)** | **0.75** | **0.76** | **0.69** | **0.78** | **0.73** | **0.68** | **0.74** | **0.69** |
| **resolution (map/model)**** | **4.0** | **3.7** | **4.3** | **3.4** | **3.5** | **4.6** | **3.5** | **3.8** |
| **PDB accession code** | **6ZSG** | **6ZSA** | **6ZSB** | **6ZSD** | **6ZSC** | **6ZSE** | **6ZSF** | **6ZS9** |
| **EMDB accession code** | **EMD-11397** | **EMD-11391** | **EMD-11392** | **EMD-11394** | **EMD-11393** | **EMD-11395** | **EMD-11396** | **EMD-11390** |
| **model composition** |  |  |  |  |  |  |  |  |
| **# atoms** | **315651** | **313935** | **312295** | **313934** | **311801** | **313955** | **312210** | **313827** |
| **# protein residues** | **14045** | **14031** | **14031** | **14031** | **14008** | **14033** | **14024** | **14223** |
| **# nucleotide residues** | **2600** | **2562** | **2523** | **2562** | **2526** | **2561** | **2526** | **2487** |
| **# GTP** | **1** | **1** | **1** | **1** | **1** | **1** | **1** | **1** |
| **# Mg ions** | **195** | **195** | **195** | **195** | **195** | **195** | **195** | **195** |
| **# Zn ions** | **7** | **7** | **7** | **7** | **7** | **7** | **7** | **7** |
| **#DOL** | **1** | **1** | **1** | **1** | **1** | **1** | **1** | **1** |
| **#QU1** | **1** | **1** | **1** | **1** | **1** | **1** | **1** | **1** |
| **B-factor (Å2)** |  |  |  |  |  |  |  |  |
| **- protein (mean)** | **105.49** | **108.07** | **95.75** | **100.13** | **53.62** | **108.78** | **82.5** | **65.8** |
| **-nucleotide (mean)** | **82.66** | **85.76** | **87.42** | **78.4** | **54.44** | **109.23** | **72.48** | **60.54** |
| **-ligand (mean)** | **165.70** | **157.9** | **121.76** | **167.37** | **80.65** | **184.12** | **145.49** | **46.73** |
| **rotamer outliers (%)** | **0.05** | **0.02** | **0.04** | **0.06** | **0.12** | **0.02** | **0.04** | **0.02** |
| **Ramanchandran (%)** |  |  |  |  |  |  |  |  |
| **- outliers** | **0.05** | **0.04** | **0.02** | **0.02** | **0.01** | **0.03** | **0.03** | **0.02** |
| **- allowed** | **4.74** | **4.58** | **4.87** | **4.52** | **4.54** | **4.56** | **4.57** | **4.62** |
| **- favored** | **95.21** | **95.37** | **95.11** | **95.46** | **95.45** | **95.41** | **95.40** | **95.36** |
| **clash score** | **5.06** | **5.01** | **5.38** | **4.83** | **4.38** | **5.53** | **4.7** | **5.14** |
| **MolProbity score** | **1.6** | **1.59** | **1.61** | **1.57** | **1.53** | **1.62** | **1.56** | **1.6** |
| **RMSD** |  |  |  |  |  |  |  |  |
| **- bonds (Å)** | **0.005** | **0.005** | **0.005** | **0.005** | **0.006** | **0.003** | **0.005** | **0.005** |
| **- angles (°)** | **0.564** | **0.543** | **0.533** | **0.55** | **0.551** | **0.528** | **0.55** | **0.545** |
