# Supplementary figures and images for: Structural basis of mitochondrial translation
Source: eLife. 2020 Aug 19;9:e58362. doi: 10.7554/eLife.58362 (PMC7438116; doi:10.7554/eLife.58362)

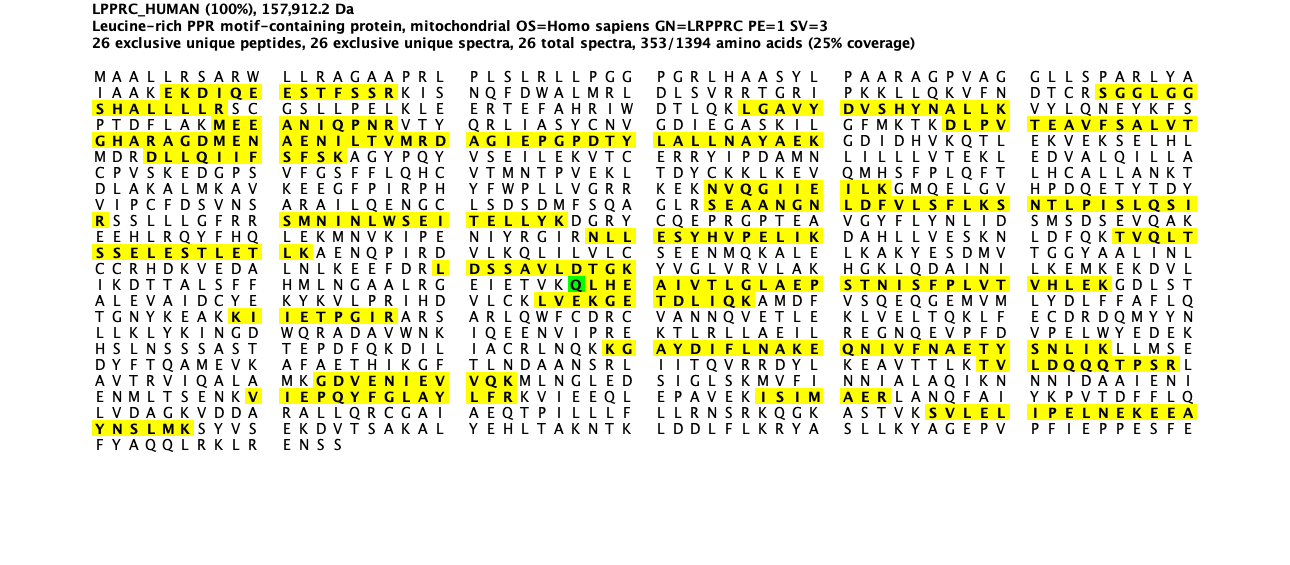


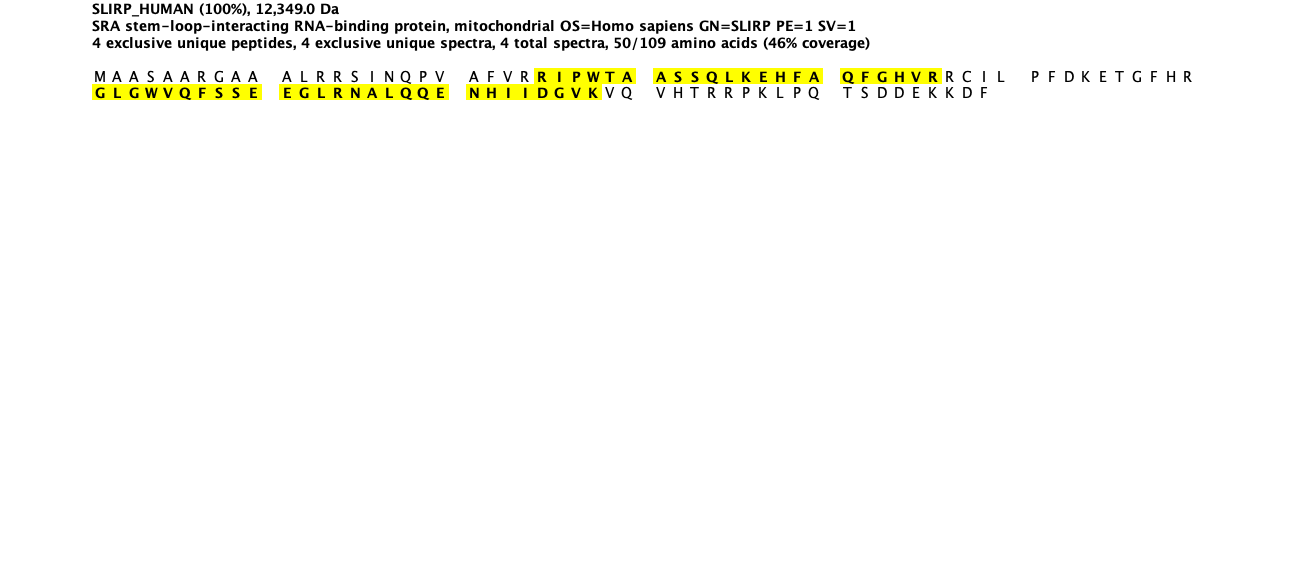

Supplement: Supplementary file 4. — The peptides identified by mass spectrometry that meet 40% minimum threshold are highlighted in yellow. Above the sequence, the protein accession number, molecular weight and protein name are shown together with the number of unique peptides, spectra and % coverage. [file elife-58362-supp4.docx]
